# The effect of preparation time and aeration rate on the properties of bulk micro-nanobubble water using hydrodynamic cavitation

**DOI:** 10.1016/j.ultsonch.2022.105965

**Published:** 2022-02-25

**Authors:** Shaoqi Zhou, Sabereh Nazari, Ahmad Hassanzadeh, Xiangning Bu, Chao Ni, Yaoli Peng, Guangyuan Xie, Yaqun He

**Affiliations:** a Key Laboratory of Coal Processing and Efficient Utilization (Ministry of Education), China University of Mining and Technology, Xuzhou 221116, China; b School of Chemical Engineering and Technology, China University of Mining and Technology, Xuzhou 221116, China; c Maelgwyn Mineral Services Ltd, Ty Maelgwyn, 1A Gower Road, Cathays, Cardiff CF24 4PA, United Kingdom; d Department of Geoscience and Petroleum, Faculty of Engineering, Norwegian University of Science and Technology, Trondheim, Norway

**Keywords:** Bulk nanobubbles (BNBs), Bulk microbubbles (BMBs), Preparation time, Aeration rate, Dissolved oxygen (DO) content, Bubble concentration and size

## Abstract

Fundamental research on bulk micro-nanobubbles (BMNBs) has grown rapidly due to the demand for their industrial applications and potential role in interfacial sciences. This work focuses on examining properties of such bubbles, including the number, concentration, zeta potential, and surface tension in water. For this purpose, BMNBs were generated by the hydrodynamic cavitation (HC) mechanism. Distilled water and air in the experiments were the liquid and gas phases, respectively. The characterization of bulk microbubbles (BMBs) and bulk nanobubbles (BNBs) were performed through focused beam reflectance measurement (FBRM) and nanoparticle tracking analysis (NTA) techniques, respectively. Zeta potential and surface tension of aqueous solutions were measured at different time and aeration rates. The results showed that aeration rate and preparation time had an important role in the properties of BNBs (concentration, bubble size, and surface charge) and BMBs (number, and bubble size). The instability of BMBs led to the rapid changes in the dissolved oxygen (DO) content in the water. The number of BMBs decreased when preparation time and aeration rate increased, but their size remained constant. By enhancing the preparation time and aeration rate, the concentration of BNBs improved first and then reduced. Additionally, the surface tension of an aqueous solution containing BNBs was significantly lower than that of pure water.

## Highlights

• Bulk micro-nanobubble solutions were prepared using a fine-bubble generator under different preparation times and aeration rates.
• The dissolved oxygen contents of bulk micro-nanobubble water under different conditions were evaluated.
• The bubble concentrations and sizes in bulk micro-nanobubble water under different conditions were evaluated.
• The relationship between the dissolved oxygen concentration and the cavitation behaviors of bubbles was discussed.

## 1 Introduction

In recent years, nanobubbles (NBs) have entered the commercial world due to their unique physical, chemical, and physicochemical properties [1], [2]. They show technological potentials in several applications, including wastewater treatment [3], flotation [4], [5], agriculture [6], nanoscope cleaning [7], [8], fuel [9], biological and medical applications [10], [11]. The development of these applications is attributed to their unique advantages, such as low ascending velocity, long longevity retention time, massive interfacial surface area, high internal gas pressure, and negatively charged surface characteristics [12].

It is widely known that NBs are gas cavities with a diameter below 1 μm, also known as ultrafine bubbles [2], [13], [14]. They can form at the solid–liquid interface and in solution, which are known as surface nanobubbles (SNBs) and bulk nanobubbles (BNBs), respectively. In SNBs, the bubbles are trapped on solid surfaces and have a diameter of between 10 and 100 nm, while in BNBs, the bubbles are dispersed in liquids, and their diameter is less than 1,000 nm [14]. With the refinement and diversification on visualization techniques, evidences for their existence have been addressed broadly in the literature [15], [16], [17], [18], [19]. For instance, Karpitschka, et al. [20] used nonintrusive optical interference-enhanced reflection microscopy to demonstrate that SNBs form in less than a few seconds after ethanol–water exchange. Also, Yasui, et al. [21] observed BNBs using transmission electron microscope without freezing. Recently, a great deal of research has been carried out using various methods for generating BNBs [12], [22], [23], [24], [25], [26], [27], [28]. Different methods have been reported for producing BNBs, including hydrodynamic cavitation (HC) [29], [30], acoustic cavitation [24], [31], organic solvent–water mixing [32], electrolysis [33], and compression-decompression of soluble gases [34]. Among these methods, cavitation is one of the most popular methods in the industry due to the low operational costs and simplicity of the equipment [35]. In addition to the generating methods, physicochemical properties of BNBs were measured using a variety of techniques such as nanoparticle tracking analysis (NTA) [36], dynamic light scattering (DLS) [29], and laser particle size analyzer (LPSA) [5].

In the last two decades, various methods were studied to produce and characterize BNBs and BMBs [12], [17], [18], [22], [37], [38]. Jin et al. [39] discovered that the size of the NBs depends on the bulk phase. They found that salt ions can adversely affect the stability of BNBs, causing them to coalesce at higher salt concentrations. Kikuchi et al. [40] applied the electrolysis method to generate BNBs. In that process, the oxygen was produced near the anode, while hydrogen was formed near the cathode, which resulted in a supersaturated gas in the solution. Then, electrolyte solutions were examined using a DLS technique [41]. Maoming et al. [42] investigated key parameters governing the median size and the volume of NBs. They discovered that bubble size grew with increasing the dissolved oxygen gas and the carbon dioxide gas concentration, the time interval, and the pressure drop within the NBs generator. Wu et al. [43] applied a baffling high-intensity agitation cell to prepare NBs with an average diameter of around 500 nm. It was found that the bubble size was significantly affected by impeller agitation speed, while the temperature and agitation duration played only an insignificant role. A study by Azevedo et al. [44] measured NB concentrations as a function of saturated pressure and surface tension using a bench system consisting of a saturator vessel and needle valve. Etchepare et al. [2] used a multiphase centrifugal pump and needle valve to generate NBs. It was discovered that the size of NBs remained constant, but numeric concentrations increased as bubble generation cycles progressed. Also, Sjogreen et al. [11] reported that NaCl concentration, temperature, and pH had an critical impact on the nano-bubble diameter. Birkin et al. [45] produced NBs by ultrasonic cavitation and measured their electrochemical properties in situ to confirm their existence. Ferraro et al. [34] used a syringe to perform successive expansion-compression cycles to generate NBs. In their experiments, they found that the concentration of NBs was improved with increasing expansion-compression cycles and then was stabilized as the dissolved gas supply diminished. Some researchers found that during the generation of BMNBs, liquid water becomes milky due to the presence of a huge amount of BMBs. Several minutes after stopping the generation, liquid water appeared transparent again since most of the BMBs have risen to the surface of the liquid. Many BNBs remained in the transparent liquid water, measured by DLS, LPSA, and NTA. The BNBs could remain stable for more than a month after their generation. However, the main problem was that it was impossible to monitor both BMBs and BNBs simultaneously [46], [47], [48].

Although the BMNBs generation methods are thoroughly studied, there are many aspects of their formation, physicochemical properties, and stabilities which remain unclear, and require a profound understanding. To this end, it is essential to measure the bubble properties at various scales by changing operating parameters. However, only a few studies have investigated BNBs characteristics as a functional of practical generating factors, so far, which is tackled in this paper. Thus, the purpose of this study is to determine how preparation time and aeration rate affect the properties of BMNBs containing water (DO content, bubble size distribution and bubble concentration). In particular, the relationship between the dissolved oxygen concentration and the cavitation behaviors of bubbles is studied.

## 2 Materials and methods

### 2.1 Materials

NaCl (AR, Sinopharm Chemical Reagent Co., Ltd) was used as a background electrolyte for the zeta potential measurements. Clean ultrapure water with a conductivity of about 18.2 MΩ·cm was produced using the Easy-2-15 (Heal Force, China) water purification system and employed for all experimental tests. All glassware utilized in this work was ultrasonically cleaned by ultrapure water thoroughly to eliminate any impurities, and then dried overnight in a vacuum drying oven prior to use. This method of cleaning ensured that the glassware was contaminant-free.

### 2.2 Methods

#### 2.2.1 Production of bulk micro-nanobubbles (BMNBs)

**Fig. 1:**
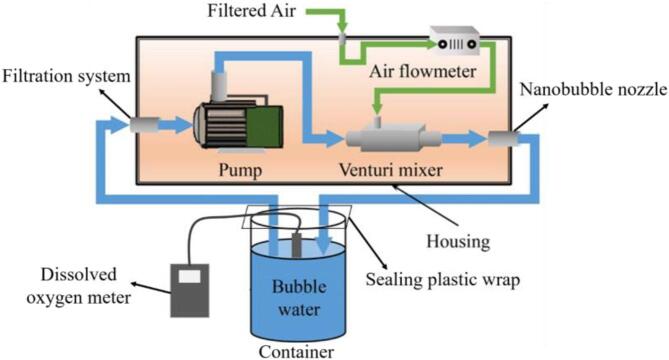
The working principle of the fine-bubbles generator.

BMNBs were produced using a fine-bubble generator (Langpai Technology Co., Ltd, Jinan, China). The generator’s working principle is shown in Fig. 1. First, ultrapure water was pumped to the venturi mixer and mixed with the gas sucked in by the self-aspirated venturi tube. The pump was cleaned up a few times to ensure no contamination within the pipes and the pump itself. The gas–liquid mixture was then pressurized and diffused to produce BMNBs. In our experiment, 1 L ultrapure water was circulated into the ultrafine-bubble generator at different preparation times to produce BMNBs with different properties. All experiments were performed in the room temperature. It is worth noting that the dissolved oxygen test was very sensitive to the external environment, which necessitates a closed testing system. Therefore, the water container was sealed with a plastic film during the test.

#### 2.2.2 DO content measurement

The DO content and temperature of water containing BMNBs were measured simultaneously using a portable dissolved oxygen meter (HQ30d, Hach, USA) equipped with a luminescent dissolved oxygen sensor (LDO10101). The sensor was calibrated before leaving factory and conducting the experiments. The experimental data was recorded during each measurement every 10 s.

#### 2.2.3 Micro-and nano-bubble size measurements

Focused beam reflectance technique (FBRM, G400, Mettler-Toledo Ltd., USA) was applied to measure the number and size distribution of micron-sized bubbles. During the test, the FBRM probe was directly inserted into the aqueous solution at a fixed angle to ensure that bubbles could easily flow through the probe window when measurement was occurred. The laser beam was emitted along the probe tube through a set of optical devices and focused on the sapphire window. The optical instrument rotated at the speed of 2 m/s to make the spot quickly scan the bubbles flowing through the window. As the focused beam scanned the entire bubble solution, a single bubble or bubble structure backscattered the laser beam to the detector. By detecting and counting the unique pulses of these backscattered light, and then multiplying the duration of each pulse by the scanning speed, the distance across each bubble, that was, the size of the bubble, could be calculated [49], [50]. The detailed information regarding the FBRM test system is described elsewhere [29], [51].

The concentration and size distribution of BNBs were acquired by a Nanoparticle Tracking Analysis (NTA) (NS 300, Malvern, Malvern, PA, USA) using a laser light source (65 mW, which was equal to 405 nm). The NTA was coupled with a 20-fold magnification microscope and a high-speed CMOS camera.

#### 2.2.4 Surface tension and zeta potential measurements

The surface tension of water containing BNBs prepared with different operating parameters was determined by the Wilhelmy Plate method using an automatic tensiometer (JK99D, Shanghai ZhongChen Digital Technic Apparatus Co., Ltd, China). After preparing water containing BNBs, the surface tension of the solution was recorded every 10 min for one-hour long.

Zeta potential measurements of prepared solutions consisting of BNBs were performed with a zeta potential analyzer (Omni, Brookhaven, USA). To conduct the measurements, first, the BMNBs solution was prepared as described earlier. Then, it was left for 10 min to eliminate the unstable BMBs. Finally, 1 mL sample solution was taken and 1 mM NaCl was added as a background electrolyte before running the zeta potential test. Three replications were performed for all the experiments.

To date, only a few methods have been developed for measuring the zeta potential of gas bubbles, but no commercial instrument is available to furnish such information. This is due to the difficulties in introducing gas bubbles into a measuring cell and controlling bubble buoyancy forces [52]. Compared to BNBs, buoyancy significantly affects the electrophoretic velocities of BMBs [53]. In particular, after the aqueous solution was left standing for 10 min, the solution was changed from milky white to clear.

## 3 Results and discussions

### 3.1 DO content

**Fig. 2:**
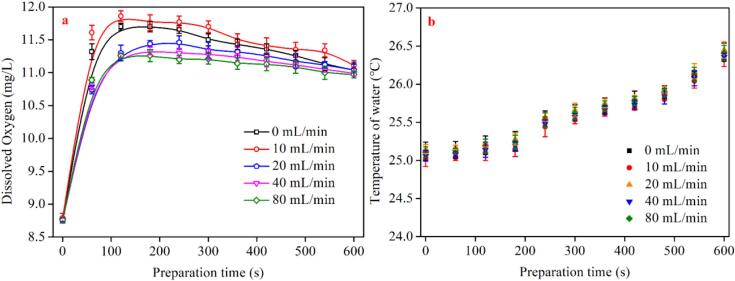
DO content (a) and water temperature (b) as a function of BMNBs preparation time at different aeration rates.

Gas nucleation and the formation of associated BNBs are dependent on the concentration of dissolved gas in a solution [54], [55]. Thus, monitoring the change in DO concentration as a function of the fine-bubbles generator's running time and aeration rate is critical for assessing the formation of BMNBs. Fig. 2a illustrates the change in the DO content of the aqueous solutions under five different aeration rates (0, 10, 20, 40, and 80 mL/min) when the preparation time varies between 0 and 600 s. As can be seen, the DO level of freshwater without operating the device is around 8.5 mg/L, which is in line with the previous reports [56]. In overall, the DO content increases rapidly as the fine-bubbles generator runs, reaches a peak of 11.3–11.8 mg/L around 100 s, and then decreases gradually. During the initial operating stages, the air is compressed into the water, which rapidly increases the DO content. Simultaneously, a large number of BMBs form, gradually turning the aqueous solution to a milky-white color. By prolonging the running time, numerous BMBs coalescence and reduce the dissolved gas content. Additionally, the temperature increase of the aqueous solution is also a reason for the decrease of DO content. Because by rising the preparation time, the temperature of aqueous solution also increases gradually (Fig. 2b). Overall, the rising temperature of aqueous solution does not exceed 1.5 ℃ within 10 min of preparation time and there is no obvious difference in the temperature of the aqueous solution prepared by different aeration rates. Therefore, in this experiment, the small increase of water temperature cannot the main reason for the change of dissolved oxygen content.

When the fine-bubbles generator runs for less than 300 s, there is a difference of around 0.75 mg/L in dissolved oxygen magnitudes between low aeration rates (0 and 10 mL/min) and relatively high aeration rate (20, 40, and 80 mL/min). However, as the duration of equipment operation increases further, the difference in dissolved oxygen between various aeration rates gradually diminishes. Such difference is less than 0.25 mg/L after the equipment operates for 600 s. The maximum value of dissolved oxygen varies with the aeration rate, and it decreases with the increase of the aeration rate. When the aeration rate was set to 0 mL/min and 10 mL/ min, the peak values of DO content are 11.72 mg/L and 11.89 mg/L, respectively, which are much higher than the values obtained under other aeration rates. Additionally, under the condition of low aeration (0 and 10 mL/min), dissolved oxygen can reach the peak faster than that of relatively high aeration rates (i.e., 20, 40, and 80 mL/min). It is interesting to note that the peak value of DO content decreased when the aeration rate was higher than 10 mL/ min. This attenuation in the peak value of DO content for a relatively higher aeration rates is related to the equilibrium between the DO content and the cavitation. By increasing the aeration rate, the DO content increases. However, an improvement in the aeration rate leads to the growth in the occurrence of cavitation events, which is directly related to the size and concentration of BMNBs. Details of the relationship between the DO content, the size and concentration of BMNBs is given in section 3.4.

**Fig. 3:**
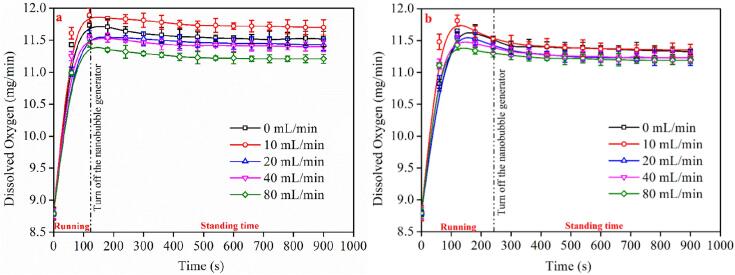
Effect of standing time on the DO level in water containing BMNBs prepared at a) 2 min and b) 4 min.

Fig. 3 displays the DO content of waters prepared at two different stabilization times (i.e., 120 s and 240 s). When the BNMBs containing aqueous solution has just been prepared, its colour appears to be milky-white. By increasing the standing time from 0 to 2 min and then 4 min, the micron-sized bubbles coalesce, float up, and finally burst, leading the aqueous solution to become clear gradually. The BNBs remain in the clear aqueous solution due to their high stability and longevity. Similar observations have also been addressed in the literature [57]. As seen in Fig. 3, the DO concentration in the solution slightly varies depending on the preparation time. The initial DO content in the aqueous solutions prepared with varying aeration rate is high when the fine bubble generator was turned off after two minutes, and there is a relatively noticeable difference in dissolved oxygen between them. Although the DO content in various aeration rates remains rather steady in a relatively short time, there are always obvious distinctions between them. The maximum DO content is achieved when the aeration rate is set to 10 mL/min, followed by 0, 20, 40, and 80 mL/min. The difference between the greatest and minimum dissolved oxygens remains about 1 mg/L even after standing for 800 s. When the fine bubble generator was turned off after 4 min, the change in the DO content of solutions with standing time follows the same trend as when the generator was turned off after 2 min. The difference is that when the preparation time is increased to 4 min, the initial DO content is lower than that of 2 min. Furthermore, when the preparation time is 4 min, the difference in dissolved oxygen between various aeration rates is small, and the dissolved oxygen difference between the highest and minimum is less than 0.25 mg/L.

Overall, after a brief period of standing (about 10 min), the concentration of dissolved oxygen in various aqueous solutions reduced only little and remained stable. During standing state, the aqueous solution gradually becomes clear from the freshly prepared milky white in a few minutes, due to the coalescence, floating, and rupture of a significant number of BMBs. It can be the reason for the modest drop in the DO content of the aqueous solution. Additionally, it also demonstrates that the absence of BMBs has little effect on the dissolved gas concentration. Thus, supersaturated dissolved gas may exist in water in a variety of forms, one of which may be BNBs. Detailed discussion of the relationship between DO content and the formation of BMNBs is presented in section 3.4.

### 3.2 Properties of MBs

**Fig. 4:**
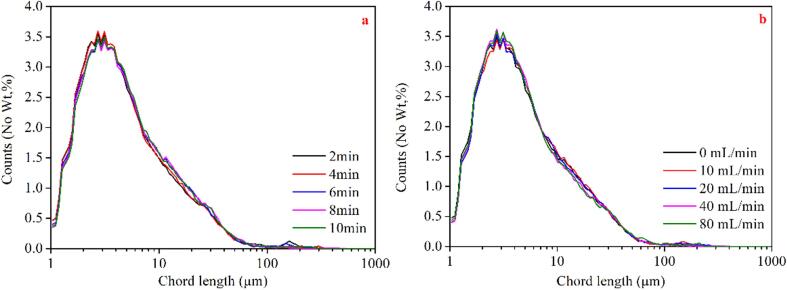
The size distribution of microbubbles at a) different preparation time while the aeration rate is 10 mL/min, and b) different aeration rate at preparation time of 6 min.

Fig. 4a and b illustrate the size distribution of BMBs generated using various operating conditions. It is obvious that the distribution of micron-sized bubbles remains constant when operational conditions vary. In other words, neither the aeration rate nor the duration of operation has any discernible effect on the size of produced bubbles. Under all conditions, the respective bubble size distribution is mostly between 1 and 100 μm, while the majority of them being less than 10 μm. The bubble size does not change during the test process because a dynamic balance is achieved between the generation and the coalescence of bubbles under different conditions.

**Fig. 5:**
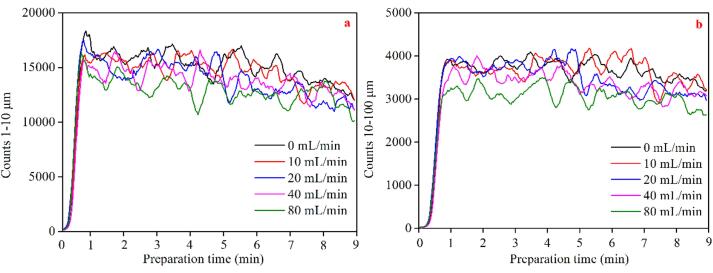
Variation of the number of microbubbles with preparation time under different aeration rates; a) sub-microbubbles with the diameter of a) 1–10 μm, and b) 10–100 μm.

Fig. 5a and b illustrate the fluctuation in the number of bubbles 1–10 μm and 10–100 μm with the equipment's running time under various aeration rates. Due to the constant generation and collapse of bubbles during the test process, each curve exhibits some volatility, but there is still a clear trend. As the duration of operation increases, the number of bubbles with the diameter of 1–10 μm grows rapidly to around 15,000–18,000 within 60 s, and then progressively diminishes. Particularly when the machine functions for 9 min, the number is reduced to less than 15,000, a reduction of approximately 30%. Compared to bubbles ranging from 1 to 10 μm, the total number of bubbles ranging from 10 to 100 µm is fewer (about 3,000–4,000). More precisely, as equipment operating time grows, the number increases dramatically to approximately 3,000–4,000 within the first 60 s. Following this, the number of bubbles in this size range remains relatively constant until the generator runs for 7 min. After 9 min of operation, the number of bubbles decreases by approximately 15%. This is because the continuous coalescence, floating, and bursting of BMBs occurs during long-term operation. Additionally, the aeration rate affects both the number of bubbles with diameters of 1–10 μm and 10–100 μm. Further, the number of bubbles in both size ranges drops as the aeration rate increases. Compared to that with aeration rate of 0 mL/min, when the aeration rate reaches 80 mL/min, the number of bubbles with a diameter of 10 μm and 10–100 μm are lowered by 18% and 15%, respectively. This is because excessive aeration rate weakens the intensity of HC (i.e., the ability of the equipment to prepare bubbles). Moreover, increased aeration rate accelerates the coalesce and rupture of bubbles, lowering the bubble number. This corresponds to the results of the DO content test.

### 3.3 Properties of water containing BNBs

#### 3.3.1 The size and concentration of BNBs

**Fig. 6:**
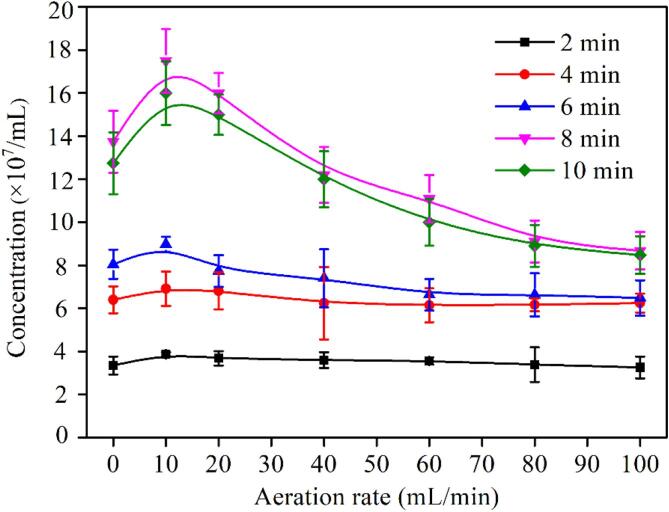
The concentration of BNBs at different operating parameters. The measurement value of particle concentration in pure water is 1.15 × 10^–7^ mL.

**Fig. 7:**
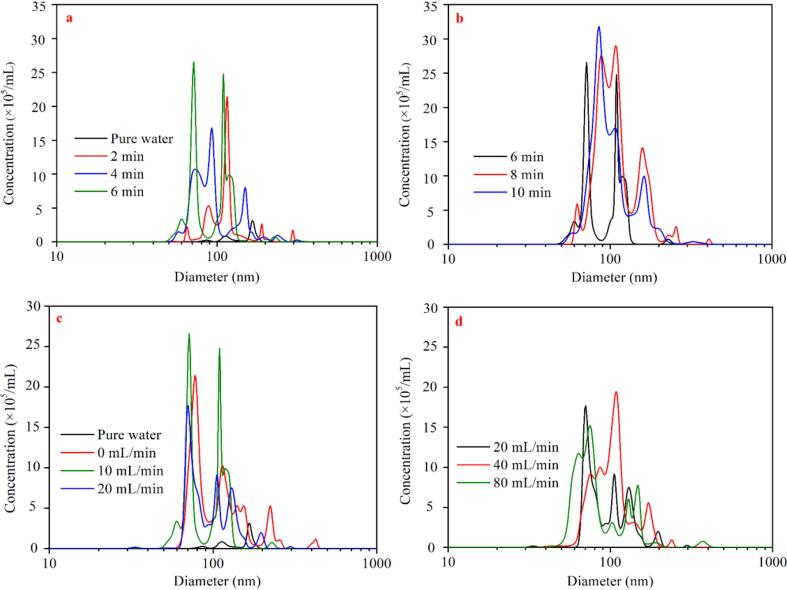
Size distribution of BNBs at different preparation time at aeration rate 10 mL/min (a-b) and at different aeration rates at preparation time 6 min (c-b).

Figs. 6 and 7 show the concentration of BNBs as a function of aeration rate and preparation time, respectively. As evidently seen from Fig. 4, Fig. 5, Fig. 6, Fig. 7, the number of BMBs, the variation in the concentration of BNBs with time, and aeration rate are entirely different. Under the same aeration rate, as the running time of the bubbles generator increases, the concentration of BNBs progressively enhances, reaching a maximum at 8 min, and subsequently decreases at 10 min. Under the same preparation time, the bubble concentration increases initially and subsequently declines as the aeration rate rises, reaching a maximum at the aeration rate of 10 mL/min. In particular, the influence of air flow rate on the BNBs concentration is insignificant when the preparation time is shorter than 6 min. The difference in concentration between BNBs at their maximum (10 mL/min) and lower concentrations is less than 1 × 10^7^/mL. However, when the preparation time is increased (>8 min), the variation ranges of BNBs concentration with aeration rate raises significantly, and the difference between the highest (10 mL/min) and lowest concentrations (100 mL/min) is approximately 7 × 10^7^/mL. This occurs because cavitation bubbles are constantly formed as the operating time of the equipment increases. Simultaneously, the shrinking and collapse of certain small BMBs result in the formation of a large number of BNBs, leading to an increase in the number of BNBs. In this regard, Jadhav and Barigou [58] found that BNBs on the order of 100 nm exist in a stable cluster form in neutral or basic medias. When the number of BNBs reaches a particular value, independent BNBs start to form BNBs clusters. Thus, the decrease in the BNB concentration can be attributed to the formation of BNBs clusters.

Fig. 7a-d respectively show the changes of BNBs as a function of preparation time and aeration rate. The nanobubble size distribution is in the range of 50–500 nm, with a peak value of approximately 100 nm. As seen, variable operational parameters (preparation time and aeration rate) have a certain impact on the bubble size distribution. When the aeration rate is held constant at 10 mL/min, the size distribution gradually shifts to the left (i.e., the bubble size decreases) as the preparation time for BNBs increases. For example, when BNBs are prepared for 2–4 min, the main peak of the size distribution is between 100 and 150 nm, however, when preparation time is prolonged to 6, 8, and 10 min, the respective main peak is between 50 and 100 nm. In comparison to the BNBs prepared in 2 and 4 min, while the main peak position of the BNBs shifts to the right at 6, 8, and 10 min (indicating that the size becomes smaller), there are several peaks at 150–200 nm, most notably at 8 and 10 min. Overall, when the preparation time for BNBs increases, the BNB size distribution decreases initially and subsequently increases. This is because as the preparation time expands, more and more small gas nuclei are formed, resulting in an abrupt increase in the number of BNBs with a diameter of 50–100 nm. When the number of BNBs reaches a particular value, the coalescence probability increases, and these BNBs coalesce to create some BNBS clusters with a size of 150–200 nm.

When the preparation time of BNBs was set to 6 min, the aeration rate also impacts its size distribution. The size of BNBs shifts to the right as the aeration rate increases. The predominant peak in the size distribution moves from 50 to 100 nm to around 100–200 nm. It may be due to the decrease in throat pressure and cavitation intensity when the aeration rate is increased. It is worth mentioning that at an aeration rate of 80 mL/min, some BNBs with a size of about 50 nm appear. This can be related to the violent collapse of micro-bubbles at a higher aeration rate, resulting in some BNBs with smaller size.

#### 3.3.2 The zeta potential of BNBs

**Fig. 8:**
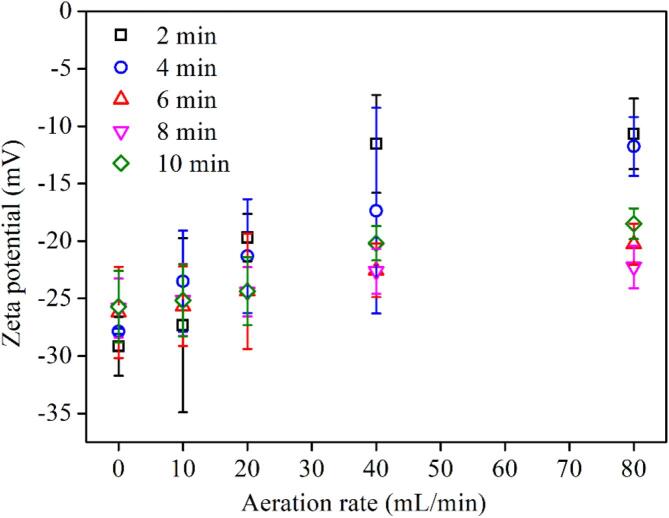
Zeta potential of BNBs at three preparation time levels (2–10 min) and aeration rates (0–80 mL/min) (0.1 mM NaCl).

The zeta potential of BNBs is a critical interfacial property. Fig. 8 exhibits the zeta potential of BNBs formed under various operational conditions. As seen, these bubbles are all negatively charged, with a potential range of −10 mV to −30 mV, which is consistent with the previous reports [17], [29]. The potential charge gradually decreases with the increase of the aeration rate. When the aeration rate is low (<20 mL/min), the preparation time has little effect on the zeta potential of bubbles. However, as the aeration rate increases (i.e., >20 mL/min), the potential charge of BNBs decreases, indicating that their electronegativity improves. As the air flow rate grows, the occurrence frequency of trainset cavitation (bubble collapse) increases, leading to the enhancement in the concentration of free radicals. HC can generate ·H and ·OH free radicals during the collapse process of bubbles [59]. Adsorbed OH^–^ and H^+^ are crucial factors influencing the gas − water interface charge; electrolyte ions are attracted to the interface by the electrostatic force and generate an electrical double layer [60]. Thus, the increase in the zeta potential of BNBs can be related to the selective adsorption of free radicals at the gas/liquid interface.

The surface charge of microbubbles under specific water conditions are similar (approximately –35 mV), regardless of their size [61] Therefore, the amount of electrical charge around the gas − water interface is the same per unit area [61]. However, Takahashi et al.’s observations concerning collapsing microbubbles over time showed that the ζ-potential increases according to the rate of shrinkage, which itself inversely proportional to the bubble size [62]. Additionally, it was demonstrated that the collapsing microbubbles generated free radicals in the absence of a dynamic stimulus, such as ultrasound or large pressure differentials [62], [63]. Different conditions can produce various sizes and concentrations of BMBs. The differences in the BMB sizes and concentrations under different condition may lead to different concentrations of free radicals after 10 min standing time of the freshly prepared bubble-containing water. Thus, future works are required to correlate the properties of BMBs (bubble size, concentration, zeta potential, and the concentration of free radicals) with the zeta potentials of BNBs.

#### 3.3.3 Surface tension of water containing BNBs

**Fig. 9:**
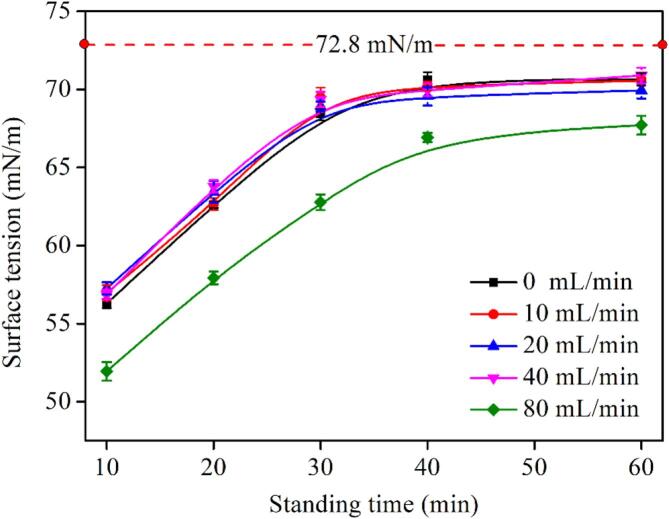
Surface tension of aqueous solutions containing BNBs under different standing times and aeration rates at preparation time 6 min.

Surface tension of solution containing BNBs at different standing times is shown in Fig. 9. As seen, after 10 min, the surface tension is 52–57 mN/m, which is significantly less than the surface tension of pure water (72.8 mN/m). The surface tension of the solution gradually increases as the standing time increases. In the first 30 min, the surface tension increases at a breakneck pace. After that, the surface tension's rising speed gradually diminishes. After one hour, the surface tension increases to approximately 70 mN/m, but it remains lower than that of pure water. Interestingly, the surface tension curves of the solutions prepared at 0–40 mL/min are nearly identical, with no discernible change, but the surface tension of the solution prepared at 80 mL/min is invariably lower than that of the aqueous solution prepared at other aeration rates. When the aqueous solution is left for 10 min, the surface tension of the solution prepared at an aeration rate of 80 mL/min is 51.94 mN/m, while its value at other aeration rates is approximately 56 mN/m. After 60 min and in 80 mL/min aeration rate, this value is approximately 68 mN/m, whereas its content at other aeration rates is approximately 70 mN/m. This can be due to the low DO content when the aeration rate is 80 mL/min.

### 3.4 Discussion

**Fig. 10:**
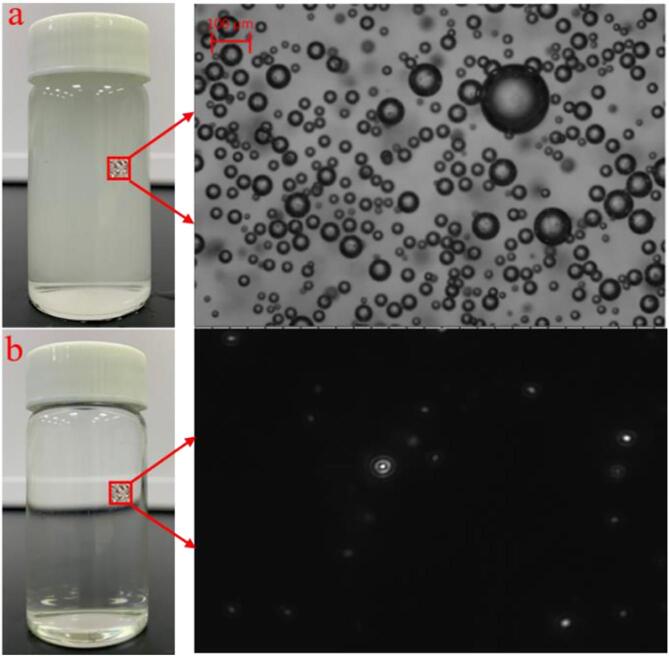
Comparison of solutions with BMNBs (a: 0 min standing time) and BNBs (b: 3 min standing time). Parameters setting: Aeration rate 10 mL/min, preparation time 6 min.

As previously established, the characteristics of aqueous solutions containing BMNBs were comprehensively studied under a variety of operating settings, with considerable differences observed between them. Due to the constant compression of gas into the water during hydrodynamic cavitation, the dissolved gas concentration in the water gradually increases, and a massive number of BMNBs are created at the same time. The presence of a significant number of BMBs imparts a milky white color to the aqueous solution (Fig. 10a). After 3 min, the BMBs in the water gradually rise to the surface and burst. At this point, the solution is transparent and contains only BNBs (Fig. 10b).

The experimental results indicate that the properties of aqueous solutions containing BMNBs are highly correlated with the properties of BMNBs. As illustrated in Figs. 3 and 5, as the preparation time increases, the DO content of water increases initially and then decreases, which is very consistent with the variation in the number of BMBs. It shows that BMBs may be an existing form of dissolved gas in water. The dissolved gas was dissipated as the microbubbles collapse and vanish. This is also why, throughout the standing time of the created BMNB aqueous solution, the DO content in the water drops.

Additionally, an intriguing phenomenon can be discovered. The number of BMBs decreases as the preparation time and aeration rate increase, however the number of BNBs gradually improves and then reduces. For BMBs, exceeding preparation time and aeration rate aggravate their coalescence. Therefore, the number of MBs gradually drops. Compared to the BMBs, the variation of BNBs’ concentration is somehow complex because of several plausible reasons. First, the cavitation nucleation behavior is affected by the aeration rate and preparation time which is a main inflicting reason on the concentration of BNBs. For example, with the increase of cavitation time, the continuous generation of cavitation nuclei increases the BNBs’ concentration. Moreover, the number of impurities produced by cavitation erosion grows as the preparation time prolongs, which may result in the increase in the number of BNBs. According to the dynamic equilibrium model, BNBs partially covered with hydrophobic material contributes to the stability of them [64]. The aeration rates affect the pressure of the venturi throat. By enhancing aeration rates, the pressure of throat is probably decreased, which may weaken the nucleation ability or cavitation intensity.

It is interesting to note that a low aeration rate is conducive to nucleation. Because under the condition of low aeration rates (10–20 mL/min), not only the cavitation threshold does not reduce too much, but also some air introduces to promote cavitation nucleation. Secondly, the generation of BNBs is related to the shrinkage and collapse of BMBs (less than 50 μm) [65]. Since the number of BMBs prepared under different operating parameters is different, this slightly affects the concentration of BNBs. For example, when the preparation time and aeration rates are high, a lot of large bubbles collapse, the number of BMBs reduces, which also means that the number of BMBs that can shrink/ collapse into BNBs might be reduced [35]. All in all, the change mechanisms of the BMNBs size and concentration are controlled by the cavitation behaviors under different operating parameters.

BMBs rupture soon after formation due to the high surface tension of pure water. As a result, BMBs have little effect on the characteristics of aqueous solution. In comparison to BMBs, BNBs have an incredible stability, as documented by a significant number of researchers. The presence of such a high concentration of BNBs may have a significant effect on the characteristics of water. Based on theoretical analysis, Yasui, et al. [57] suggested that a liquid film is more easily ruptured by the presence of BNBs at the liquid surface, which reduces the value of surface tension. However, more systematic studies should be performed to discover the influencing mechanism of BNBs on the surface tension of pure water in future. Some studies have shown that, the surface tension is strongly dependent on the initial quantity of air dissolved in the water sample. The variation in the surface tension of an aqueous solution are thought to be related to a pronounced influence of the presence of polarizable molecules (O_2_, N_2_) in the local molecular organization of the interfacial region of the water/air system [66]. The dissolved gas in supersaturated aqueous solution continually diffuses from the bulk phase to the interface during standing, and the gradual decrease in dissolved gas in aqueous solution may be one of the reasons for the dynamic change in surface tension.

## 4 Conclusions and future works

The properties of aqueous solutions containing BMNBs are highly correlated to the physical and chemical characteristics of generated fine bubbles. To this end, size and number/concentration of both BMBs and BNBs were studied as a function of aeration rate (0, 10, 20, 40 and 80 mL/min) and preparation time (0–10 min). To sum up the results, the following conclusions were drawn and highlighted below:

- Under various operating conditions, there was a strong association between the dissolved gas concentration and BMNBs, indicating that BMNBs were a form of dissolved gas in water. However, in comparison to BNBs, BMBs instability was more likely resulted a rapid change in the dissolved gas concentration of water.
- The behavior of two distinct bubble scales to operational conditions, on the other hand, was quite diverse. The number of BMBs was reduced as preparation time and aeration rate was raised, but their size remained constant. However, changes in the properties of BNBs were more complicated.
- By increasing preparation time and aeration rate, the concentration of BNBs grew to a peak value and then decreased. Later, the size of BNBs decreased initially and then increased as preparation time increased, and marginally increased as aeration rate increased.
- The electronegativity of BNBs was reduced as the aeration rate increased.
- The surface tension of an aqueous solution containing BNBs was significantly lower than that of pure water, and it steadily increased as the standing time increased.
- The work leads to a better understanding of the effect of operating parameters on the properties of BMNBs, which will help in expanding their application in other domains such as atmosphere, mining, and medicine.

### CRediT authorship contribution statement

**Shaoqi Zhou:** Methodology, Writing – original draft, Visualization. **Sabereh Nazari:** Writing – original draft, Writing – review & editing. **Ahmad Hassanzadeh:** Writing – original draft, Writing – review & editing. **Xiangning Bu:** Supervision, Conceptualization, Methodology, Writing – original draft, Writing – review & editing, Funding acquisition. **Chao Ni:** Investigation, Funding acquisition. **Yaoli Peng:** Investigation. **Guangyuan Xie:** Supervision, Investigation, Funding acquisition. **Yaqun He:** Investigation.

## Declaration of Competing Interest

The authors declare that they have no known competing financial interests or personal relationships that could have influenced the work reported in this paper.
